# Millimeter-scale topography facilitates coral larval settlement in wave-driven oscillatory flow

**DOI:** 10.1371/journal.pone.0274088

**Published:** 2022-09-12

**Authors:** Mark A. Levenstein, Daniel J. Gysbers, Kristen L. Marhaver, Sameh Kattom, Lucas Tichy, Zachary Quinlan, Haley M. Tholen, Linda Wegley Kelly, Mark J. A. Vermeij, Amy J. Wagoner Johnson, Gabriel Juarez

**Affiliations:** 1 Department of Mechanical Science and Engineering, University of Illinois at Urbana-Champaign, Urbana, IL, United States of America; 2 Institute for Genomic Biology, University of Illinois at Urbana-Champaign, Urbana, IL, United States of America; 3 Department of Physics, University of Illinois at Urbana-Champaign, Urbana, IL, United States of America; 4 CARMABI Foundation, Piscaderabaai z/n, Willemstad, CW; 5 Department of Microbiology, Raboud University, Nijmegen, NL; 6 Scripps Institution of Oceanography, University of California, San Diego, La Jolla, CA, United States of America; 7 Department of Freshwater and Marine Ecology, Institute for Biodiversity and Ecosystem Dynamics, University of Amsterdam, Amsterdam, NL; 8 Carle Illinois College of Medicine, University of Illinois at Urbana-Champaign, Urbana, IL, United States of America; University of Guam, GUAM

## Abstract

Larval settlement in wave-dominated, nearshore environments is the most critical life stage for a vast array of marine invertebrates, yet it is poorly understood and virtually impossible to observe *in situ*. Using a custom-built flume tank that mimics the oscillatory fluid flow over a shallow coral reef, we isolated the effect of millimeter-scale benthic topography and showed that it increases the settlement of slow-swimming coral larvae by an order of magnitude relative to flat substrates. Particle tracking velocimetry of flow fields revealed that millimeter-scale ridges introduced regions of flow recirculation that redirected larvae toward the substrate surface and decreased the local fluid speed, effectively increasing the window of time for larvae to settle. Regions of recirculation were quantified using the *Q*-criterion method of vortex identification and correlated with the settlement locations of larvae for the first time. In agreement with experiments, computational fluid dynamics modeling and agent-based larval simulations also showed significantly higher settlement onto ridged substrates. Additionally, in contrast to previous reports on the effect of micro-scale substrate topography, we found that these topographies did not produce key hydrodynamic features linked to increased settlement. These findings highlight how physics-based substrate design can create new opportunities to increase larval recruitment for ecosystem restoration.

## Introduction

The recruitment of pelagic larvae is a critical step in the life cycle of many sessile marine invertebrates, and for reef-building corals, the pervasive failure of natural recruitment processes over the past 50 years has compounded global coral reef losses [1–3]. During this critical life stage, swimming larvae can spend days to weeks in the water column and be transported hundreds of meters to kilometers before encountering a suitable location in which to attach and settle [4]. However, because larval dispersal and settlement take place over vast spatial and temporal scales, it is extremely difficult for researchers to observe these processes as they happen in nature; larval dispersal and settlement are consequently referred to as a “black box” [5, 6]. In turn, this knowledge gap impedes the restoration of marine ecosystems, such as coral reefs, through substrate and habitat engineering [7–9].

The effectiveness of coral restoration methods relies on mimicking or improving upon natural reef environments and their properties, including the biological and physical factors that aid larval navigation, settlement, and survival [10]. Efforts to understand and promote larval settlement in particular have focused primarily on the biological cues that facilitate the transition from pelagic dispersal to benthic settlement. For example, coral larvae settle in the presence of benthic organisms such as crustose coralline algae (CCA) [11–13], bacterial biofilms [14–16], and individual organic molecules produced by CCA and bacteria [11, 17–20]; these biological cues are now commonly used to promote coral settlement in larval propagation and reef restoration. Several physical settlement cues have also been identified for coral larvae, although these have been less widely studied or applied for coral propagation and restoration. For example, coral larvae swim toward the acoustic signature of a reef [21], avoid areas with relatively high ultraviolet radiation [22], and select settlement locations based on substrate color [23] and substrate topography [24, 25]. Focusing on topography-based settlement preferences, larvae have generally been observed to settle within topographical features close to their size, which are thought to maximize their number of attachment points (i.e., Attachment Point Theory) [25–27]. However, this rationale does not fully account for the effects of flow near surfaces, especially when the prevailing hydrodynamic forces are much stronger than larval swimming and adhesion abilities.

Indeed, fluid motion is critical to the maintenance and growth of benthic marine ecosystems. Benthic boundary layer (BBL) flows, i.e., the fluid motion directly above benthic surfaces, influence the exchange of materials and resources between the water column and the benthic zone. For instance, these flows affect nutrient uptake [28, 29], gas exchange [30, 31], surface pH [32], and larval transport and settlement [33, 34]. BBL flows are determined by the fluid-structure interactions between the viscous flow profile of water and the benthic topography [35] and have characteristic fluid regions with velocity gradients (e.g., shear, strain) and recirculation (e.g., fluid rotation and vorticity). Shallow coral reefs, in particular, experience a range of dynamic BBL conditions influenced by both reef topography and strong unidirectional currents and oscillatory wave action [36]. These fluctuating flow structures produce forces and torques on marine organisms that affect their transport, their settlement, and whether they behave as active or passive particles [37]. In contrast to these dynamic natural environments, many lab-based settlement experiments and tests of restoration substrates are conducted under static or near-static conditions [25, 38]. While these approaches have produced some key developments in restoration ecology, they provide incomplete information about natural recruitment mechanisms and opportunities for further innovation in reef-site interventions.

Here, we demonstrate the potential for millimeter-scale substrate topography to facilitate larval settlement by modifying BBL flows in the absence of external biochemical cues. Millimeter-scale ridges produce boundary layers with regions of recirculation and low velocity even in wave-driven flows much faster than larval swimming speeds. In experiments performed using a custom-built, oscillatory flume tank, ridged substrates that produced these boundary layer features received more than 10-fold greater settlement of two species of Caribbean broadcast-spawning coral larvae than compared to flat substrates. Experiments were performed in the absence of external biochemical settlement cues, such as CCA, in order to better isolate the effect of hydrodynamic forces on settlement. The underlying settlement mechanisms were elucidated by computational fluid dynamics coupled with an agent-based model of swimming larvae. These simulations demonstrated that the unique BBLs generated over millimeter-scale ridged surfaces direct larvae toward substrates and increase the opportunity for settlement by allowing larvae to be active for longer periods of time than compared to over substrates with micro-scale ridges. Our findings suggest that while ecologically relevant flows generally make settlement more difficult, the presence of millimeter-scale topography can facilitate settlement in an otherwise unfavorable hydrodynamic environment.

## Materials and methods

### Gamete collection and Larval rearing

Gametes were collected from the hermaphroditic Caribbean broadcast-spawning corals *Diploria labyrinthiformis* (Grooved Brain Coral) and *Colpophyllia natans* (Boulder Brain Coral) at Playa Zakitó, Curaçao (also known as Water Factory; 12°6’34” N, 68°57’18” W). Egg-sperm bundles were collected from 7 *D*. *labyrinthiformis* colonies and 4 *C*. *natans* colonies at a depth of 5–10 m. Larvae were reared following previously-published methods [39–42] which are also summarized in the Supplementary Information. All larval rearing steps and experiments were performed with 0.5 μm filtered seawater (FSW; spun polypropylene sediment filters, sequential pore sizes of 50 μm, 20 μm, 5 μm, and 0.5 μm, H_2_O Distributors, Marietta, GA). *D*. *labyrinthiformis* larvae were approximately spherical with a diameter of ~300 μm [42] while *C*. *natans* larvae were a prolate spheroid with a semi-major axis length of ~200 μm and a semi-minor axis length of ~150 μm.

### Flume tank design and operation

**Fig 1:**
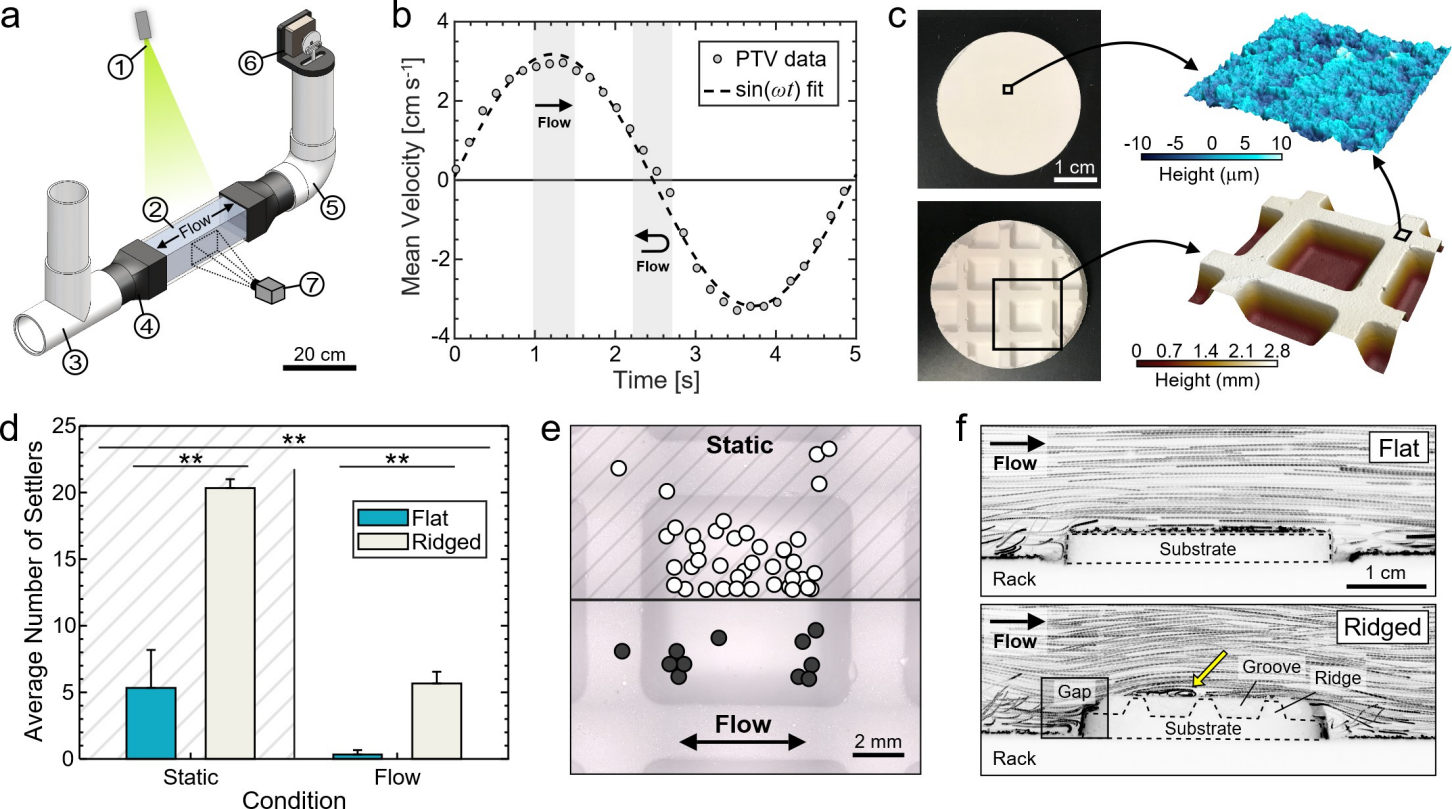
Settlement of coral larvae onto flat and ridged substrates in oscillatory flow. (a) Schematic of the oscillatory flume tank and particle tracking system. Major components include (1) laser sheet, (2) acrylic viewing section, (3) PVC T-socket, (4) custom 3D-printed PVC-to-acrylic connectors, (5) PVC elbow, (6) motor and piston assembly, and (7) high-speed camera. (b) Spatially-averaged fluid velocity in the central viewing section of the flume obtained from particle tracking velocimetry measurements (PTV; circles) over a full oscillation period. The grey highlighted sections correspond to the phases of peak flow (*straight arrow*) from left to right and the turning point (*curved arrow*) from rightward to leftward flow. (c) Photographs of the flat and ridged CaCO_3_-based settlement substrates and 3D laser confocal maps showing the micro-scale topography of both substrate types (*top*) and the millimeter-scale ridges of the ridged substrates (*bottom*). (d) Larval settlement data on flat and ridged substrates in static and oscillatory flow conditions (**: *p* < 0.001; post-hoc Tukey HSD). (e) Top view of a ridged substrate illustrating larval settlement locations in static (*top*, *n* = 40) and flow (*bottom*, *n* = 11) conditions overlaid onto a single ridged section. (e) Tracer particle pathlines during peak flow above flat (*top*) and ridged substrates (*bottom*). A region of flow recirculation above the ridged substrate is identified (*yellow arrow*).

Larval settlement experiments and flow visualization measurements were conducted in a custom-built, U-shaped flume tank (Fig 1A) with the following components: (1) laser sheet, (2) acrylic viewing section, (3) PVC T-socket, (4) custom 3D-printed PVC-to-acrylic connectors, (5) PVC elbow, (6) motor and piston assembly, and (7) high-speed camera. The central viewing section had a square cross section with dimension of 9.1 × 9.1 cm^2^. All experiments were run using a sinusoidal oscillatory flow with a period between 5–6 s and a peak mean velocity between 3–5 cm s^-1^ (Fig 1B); these parameters were selected to mimic the wave-dominated conditions often encountered in shallow coral reef habitats used in other studies of larval settlement [34, 43–46]. The Reynolds number, defined as Re = *UL*/*ν*, ranged from 3000–4400 at the peak flow phase, which is in the transitional flow regime. Here, *U* is the mean peak flow speed, *L* is the hydraulic diameter of the flume, and *ν* is the kinematic viscosity of the seawater. A power spectral density analysis of the velocity fluctuations at 30 mm above the substrate showed that less than 2% of the total energy was contained in turbulent fluctuations at the highest Reynolds number. Additional information on flume construction and operation is provided in the S1 File.

### Settlement substrate fabrication

Surface topography was controlled by manufacturing calcium carbonate (CaCO_3_) settlement substrates from an un-aged lime mortar (see Supplementary Information) [47]. Substrates were prepared with either a flat top surface or with ridges that were 2.5 mm tall and spaced 7.5 mm apart edge-to-edge (spacing to height ratio of 3, Fig 1C). The final surface topography of both substrate types was characterized with a 3D laser scanning confocal profilometer (Keyence VK-X1000; Fig 1C). In addition to the presence or absence of millimeter-scale ridged features, both substrate types had inherent microscale topography with a mean roughness of 1.88 ± 0.23 μm and a maximum peak height of 13.20 ± 1.81 μm (mean ± standard deviation; *n* = 3). For larval settlement experiments, a 3D-printed rack was designed to hold eight individual settlement substrates in the transparent acrylic section of the flume. Substrates were held in a 2 × 4 array, with a row of four flat substrates and four ridged substrates each oriented parallel to the oscillating flow (S6 Fig in S1 File).

### Flow visualization

The flow fields generated within the flume and over the substrates were characterized by particle tracking velocimetry (PTV). A laser pointer (<5 mW; 405 nm wavelength) was positioned above the transparent acrylic section of the flume and directed through a plano-concave cylindrical lens to create a vertical laser sheet along its length (Fig 1A). Fluorescent green polyethylene microspheres (Cospheric, 250–300 μm diameter, 0.99–1.01 g cm^-3^) were used as tracer particles. A scientific CMOS camera (Ximea) with a Nikkor macro lens (Nikon) was positioned perpendicular to the laser sheet to capture the movement of the particles over time. Videos were recorded at 90 frames s^-1^ with a spatial resolution of 31 μm per pixel. For each flow phase (i.e., *peak* flow and *turning point* flow), PTV data were averaged over 40 frames collected across the respective phase (Δ*t* = 0.44 s). Representative videos of the flow over flat and ridged substrates in the 3D-printed rack in the flume can be found in the S1 File and S1 and S2 Videos. A separate, 3D-printed substrate with features matching the 2D profile of the ridged CaCO_3_ substrates was used to visualize the flow field between each millimeter-scale ridge (S7 Fig in S1 File and S5 Video).

### Quantitative flow analysis and identification of recirculation

Quantitative measurements of boundary layer flows (rotation, strain, and velocity) were obtained from the particle tracking velocimetry (PTV) data and were examined in relation to observed patterns of larval settlement. Regions of recirculation near substrate surfaces were also identified. Vorticity magnitude is commonly used to identify recirculation in flows, but, especially near surfaces, it does not distinguish between actual recirculation and parallel shear flows [48, 49]. To identify regions of true recirculation, vortex cores in instantaneous velocity fields were located using the *Q*-criterion metric [50, 51]. The *Q*-criterion is defined as *Q* = 1/2(|Ω|^2^ - |*S*|^2^) > 0, where Ω is the vorticity tensor and *S* is the rate-of-strain tensor. A positive value of *Q* corresponds to a region where the local rotation exceeds the local strain, a characteristic of flow recirculation. A negative value of *Q* corresponds to a region in the flow field where the local strain exceeds the local rotation, such as in laminar shear flow over a flat surface. This differentiation of fluid rotation due to recirculation (i.e., in relatively low strain regions) from rotation due to shear (i.e., in relatively high strain regions) allowed direct investigation of the role of flow recirculation in larval settlement. In practice, a non-zero threshold, *Q*_*thresh*_, must also be used to accurately identify true recirculation. Here, *Q*_*thresh*_ is defined as the standard deviation (SD) in *Q* to account for spurious experimental results, often encountered when tracking particles near surfaces.

### Larval settlement experiments

For all settlement experiments, 750–1000 larvae were added into the flume tank at a density of 50–70 larvae L^-1^ FSW. The flumes were located in a room where the temperature was maintained at 27.5 ± 1.0°C to match the temperature of the reef where gametes were collected. The motor drive and piston were set to produce a flow speed (3–5 cm s^-1^) and period (5–6 s) that mimicked observed reef conditions [34, 43–46]. Control replicates without an applied flow (i.e., static conditions) were also run. Experiments were scored after 1 day for *D*. *labyrinthiformis* and after 2 days for *C*. *natans* (because the latter were slower to settle). During scoring, the location (substrate top, side, bottom, or rack) and number of attached larvae, regardless of shape, were recorded for each substrate type (S1 Fig in S1 File). *D*. *labyrinthiformis* experiments were repeated three times (*n* = 3), and *C*. *natans* experiments were repeated four times (*n* = 4) for both flow and static conditions. Additionally, the exact locations of settlers were mapped for two *D*. *labyrinthiformis* runs. To account for random effects related to substrate position, the flume was kept in a temperature-controlled room and the location of the flume within the room and the placement of the flat and ridged substrates were alternated for each replicate run.

### Agent-based simulation of larval settlement

A finite-element model of the flume was developed in COMSOL Multiphysics software to simulate the boundary layer velocity fields that formed over different substrate topographies under an applied oscillatory period of 5.5 s and a mean free stream velocity of 4.5 cm s^-1^ at peak flow. In addition to the flat and 2.5 mm tall ridged topographies, we also simulated 0.25 tall ridges with the same spacing to height ratio of the 2.5 mm ridges (a ratio of 3). The boundary conditions were set as follows: periodic side boundaries, a symmetry condition on the top boundary, and a no-slip condition on the bottom substrate. The simulation used a triangular mesh that increased in density close to the no-slip substrate. The flow simulation was validated for mesh and time-step convergence and periodicity (S8 and S9 Figs in S1 File) and the resultant velocity fields were imported into MATLAB to compute larval trajectories in the boundary layer flows that developed. Imported velocity fields were gridded with a grid spacing of 0.05 mm. Larvae were modeled as neutrally-buoyant ellipses with a constant swimming speed along the direction of their major axis. To avoid biasing settlement by initial conditions, 19,200 larvae were simulated for each substrate topography with individual starting conditions (30 initial positions, 32 initial orientations, and 20 initial times in a period). Their translational motion was calculated using the equation:

$$\dot{r}=U+u_{l}\hat{n}$$ (1)

where $\dot{r}$ is the total larval velocity, ***U*** is the local flow velocity, *u*_ℓ_ is the larval swimming speed, and $\hat{n}$ is the orientation of the larva’s major axis. Their rotational motion was calculated using the equation:

$$\dot{\theta }=\frac{\omega_{z}}{2}+\alpha \hat{g}∙E\hat{n}$$ (2)

where $\dot{\theta }$ is the total larval angular velocity, *ω*_*z*_ is the vorticity, *α* is the shape parameter, ***E*** is the symmetric rate of strain tensor, and $\hat{g}$ is the unit vector along the larva’s minor axis. The shape parameter is defined as: $\alpha =\left(1-{\left(b/a\right)}^{2}\right)/\left(1+{\left(b/a\right)}^{2}\right)$, where *a* is the semi-major axis and *b* is the semi-minor axis. We simulated larvae with a representative size of *a =* 0.25 mm and *b =* 0.15 mm (corresponding to a length of 0.5 mm and a width of 0.3 mm) and a species-averaged swimming speed of 3 mm s^-1^ (calculated from measured horizontal, downward, and upward swimming speeds of *n* = 9 species of larvae) to ensure the generality of modeling results (S1 Table in S1 File) [34]. Agent-based larval simulations used periodic side boundaries, a rigid bottom substrate, and an open top boundary. Trajectories were computed until either (1) larvae encountered a surface while their total speed ($\dot{r}$) was lower than the larval swimming speed plus one standard deviation, at which time they were considered to be settled on the substrate, or (2) until larvae exited the top of the simulation domain. A time step of 0.01 s was chosen because it was a small fraction (0.18%) of the oscillation wave period and even in the highest flow speed regions larval displacement was a maximum of 1.5 larval body lengths.

### Statistical analysis

Significant differences in settlement between substrate types under different flow conditions were determined with two-way analysis of variance (ANOVA) and post-hoc Tukey’s Honestly Significant Difference (HSD) tests using a significance level (α) of 0.05. The number of settled larvae in a particular location was converted into a proportion of the total larvae added (*n* = 1000) and normalized using an angular transformation (arcsine square root) prior to significance testing [52]. The flow condition (static *versus* flow) and substrate topography (flat *versus* ridged) were assumed to be fixed variables, and the test had 11 degrees of freedom (df). For both species, data from larval settlement onto the substrates and into the gap regions were also recast according to the calculated *Q*-criterion value above each location [52]. Here, the *Q*-criterion (below or above *Q*_*thresh*_) and species (*D*. *labyrinthiformis* or *C*. *natans*) were treated as fixed factors for two-way ANOVA (df = 13). Settlement events were assumed to have a negligible effect on the supply of available larvae because less than 10% of all larvae settled in each experimental run. Differences in settlement in the agent-based larval simulations were analyzed with one-way ANOVA and post-hoc Tukey HSD tests assuming substrate topography to be a fixed variable (df = 1919).

## Results

### Settlement of coral larvae onto engineered substrates in oscillatory flow

Larvae of the corals *Diploria labyrinthiformis* (Grooved Brain Coral) and *Colpophyllia natans* (Boulder Brain Coral) were subject to static conditions and controlled oscillatory flow (3–5 cm s^-1^ mean peak velocity and 5–6 s period) over engineered settlement substrates with either flat surfaces or millimeter-scale ridges (Fig 1B and 1C). Focusing first on *D*. *labyrinthiformis* larvae due to the low absolute settlement of *C*. *natans* (discussed below), both flow and substrate topography had significant effects on larval settlement (Fig 1D). *D*. *labyrinthiformis* settlement was 4.3 times lower in oscillatory flow compared to in static conditions (*p* = 0.0002, F = 34.5). However, in both flow and static conditions, more larvae settled onto the ridged substrates than onto the flat substrates (Fig 1D; *p* = 0.0001, F = 42.2). In particular, settlement was nearly zero on flat substrates in oscillatory flow (0.33 settlers per run, *n* = 3 runs; Fig 1D).

The importance of substrate topography in facilitating larval settlement was also illustrated by the settlement locations of individual *D*. *labyrithiformis* larvae (Fig 1E). Settlement locations in static conditions (*n* = 40 settlers) and oscillatory flow (*n* = 11 settlers) were mapped onto a single section of a ridged substrate for comparison. In both cases, >87% of larvae settled between the ridges rather than on the tops of the ridges. However, settlement patterns in the spaces between the ridges were markedly different depending on flow. In static conditions, larvae settled uniformly across these areas (Fig 1E, *upper panel*) while in oscillatory flow, 90% of larvae settled within 1 mm of the base of the ridges (Fig 1E, *lower panel*). In particular, settlement was concentrated near the ridges that were perpendicular to the oscillatory flow.

An unexpected result further highlighted the importance of substrate topography for larval settlement: 67% of *D*. *labyrinthiformis* larvae (*n* = 190) and 74% of *C*. *natans* larvae (*n* = 26) settled not onto the tops of substrates, but within the cryptic spaces that were formed by gaps between the 3D-printed holding rack and the substrates (due to slight size differences created during fabrication; Fig 1F, S1 Fig in S1 File). In particular, the settlement of *C*. *natans* larvae onto the tops of substrates alone was too low to distinguish any settlement trends without considering the settlement into these cryptic spaces. Overall, we found substantial evidence that substrate topography drives coral larval settlement, especially in wave-dominated, oscillatory flow, regardless of whether the topography was the ridges on the substrates or the gaps between the substrates and their holding rack. Based on these striking results, we conducted a detailed investigation of the boundary layer flow in the flume to visualize and quantify the hydrodynamic mechanisms underlying the preferential settlement of larvae onto ridged substrates and into cryptic habitats.

### Ridged substrates create recirculating boundary layer flow

Qualitative differences in the flow fields over both substrate types were visualized by tracking fluorescent tracer particles over a full oscillatory period and plotting the resulting data for two characteristic phases of the flow period: peak rightward flow (“peak flow”, Fig 1B, *straight line region*) and the turning point from rightward to leftward flow (“turning point flow”, Fig 1B, *curved line region*). Due to the symmetry of the sinusoidal flow and the substrate rack, these phases are symmetric with the peak leftward phase and the opposing turning point, respectively.

Boundary layer flows differed according to substrate topography. During the peak flow phase, particle pathlines revealed that the bulk flow was laminar far above both substrate types (>>1 mm; Fig 1F) and, for flat substrates, the boundary layer flow was also laminar near the substrates (<1 mm). However, for ridged substrates, particle pathlines revealed regions of fluid recirculation between the ridges (Fig 1F, *yellow arrow*). During the turning point phase, regions of recirculation also formed above the gaps between the 3D-printed rack and the sides of the substrates (S1 and S2 Videos), but no recirculation was observed directly above the flat substrates.

### Topography-induced flow recirculation corresponds to coral settlement location

**Fig 2:**
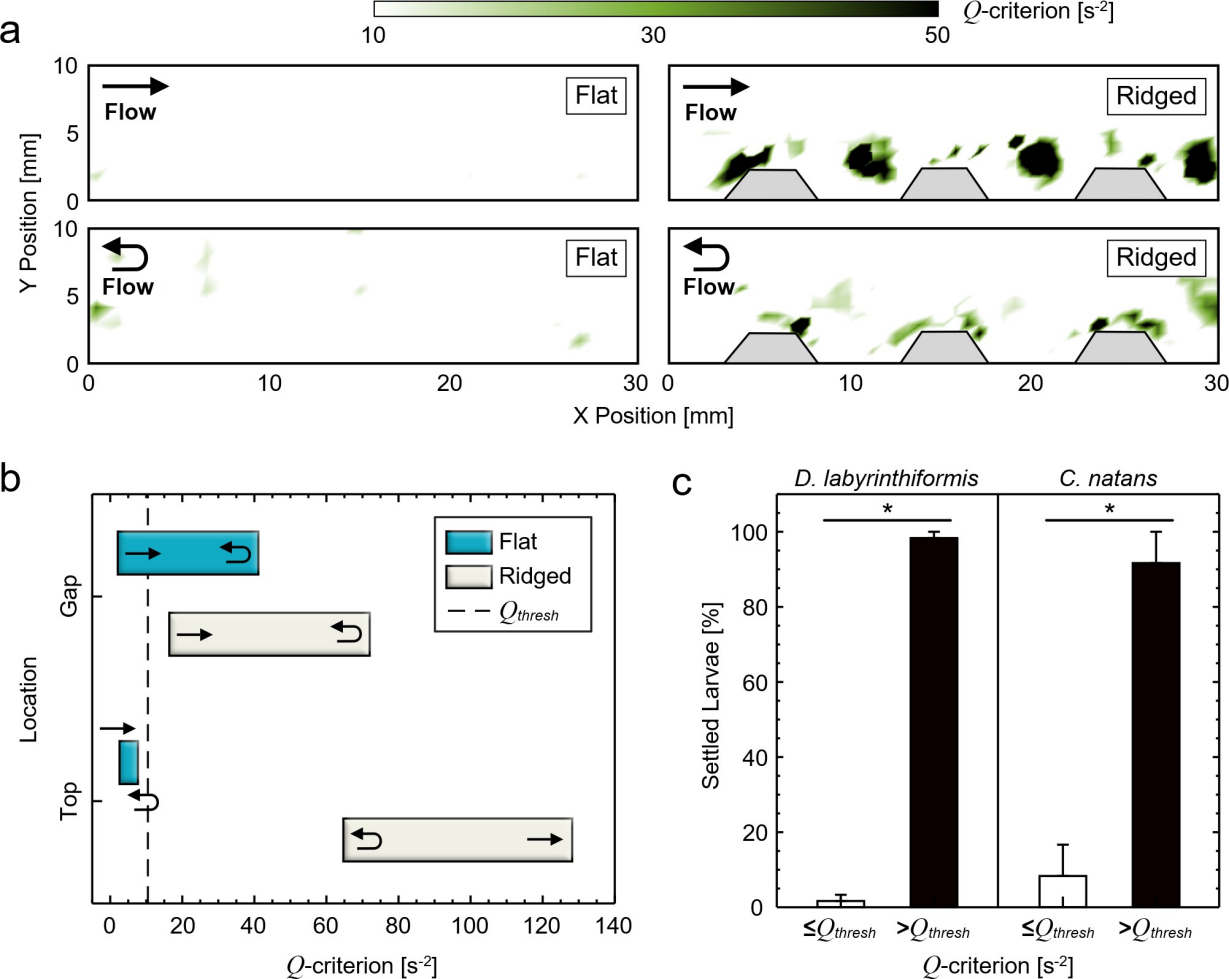
Millimeter-scale ridges generate regions of flow recirculation that influence larval settlement. (a) Regions of flow recirculation identified over flat (*left*) and ridged (*right*) substrates during peak (*top*) and turning point (*bottom*) flow using the *Q*-criterion metric. (b) Range plot of the maximum *Q*-criterion values over flat and ridged substrates and gap regions during a full period of oscillation. The dotted line is the average standard deviation in *Q*-criterion across all measurements, which was used as the threshold for the identification of vortex structures (*Q*_*thresh*_). (c) Larval settlement data under a flow region with a *Q*-criterion greater than or less than *Q*_*thresh*_ for *D*. *labyrinthiformis* (*left*) and *C*. *natans* (*right*). Settlement is presented as the mean percent of total settlers in each experimental run and the error bars represent the standard error of the mean. There was a significant difference in the settlement between regions above and below the *Q*_*thresh*_ for both coral species (*: *p* < 0.01; post-hoc Tukey HSD).

Quantitative parameters extracted from particle tracking velocimetry (PTV) of fluorescent tracer particles were used to calculate the time- and spatially-resolved *Q*-criterion and identify regions of recirculation (see Methods). For flat substrates, no recirculation was observed during the peak flow phase (Fig 2A, *top*). In contrast, for ridged substrates, regions of flow recirculation with *Q*-criteria greater than *Q*_*thresh*_ were observed between the ridges. Vortex structures were clearly seen forming and detaching from the trailing edges of each ridge (S3 Video). At the turning point of the oscillatory period, the reversal of fluid motion produced small, short-lived regions of rotation over flat substrates. However, much greater recirculation was observed during the same phase over the ridged substrates (Fig 2A, *bottom*).

Local substrate topography affected the maximum *Q*-criterion value that occurred during the flow oscillation period (Fig 2B). Above flat substrates, the *Q*-criterion always remained lower than the threshold value, indicating that these regions were dominated by fluid strain. However, above ridged substrates, *Q*-criterion values were always much larger than the threshold value, indicating that these regions were dominated by flow recirculation. Here, *Q*-criterion values ranged from 65 s^-2^ at the turning point up to 130 s^-2^ during peak flow (Fig 2B). *Q-*criterion values greater than *Q*_*thresh*_ were also observed above the gaps between the substrates and the 3D-printed rack (S2 Fig in S1 File); here, the values reached up to 40 s^-2^ and 70 s^-2^ for flat and ridged substrates, respectively (Fig 2B).

Remarkably, when comparing all settlement locations of both *D*. *labyrinthiformis* and *C*. *natans* larvae in the context of the *Q-*criterion data, larval settlement was overwhelmingly concentrated on surfaces directly under flow regions where the *Q-*criterion was greater than *Q*_*thresh*_ at some interval during the flow period (Fig 2C). Indeed, when all larval settlement locations from both species were analyzed according to their corresponding *Q*-criterion, >98% of *D*. *labyrinthiformis* larvae (*n* = 64) and >91% of *C*. *natans* larvae (*n* = 33) settled under a flow region with a *Q*-criterion > *Q*_*thresh*_ (*p* = 0.0013, F = 18.5; Fig 2C).

### Substrate topography extends the settling window near substrates

**Fig 3:**
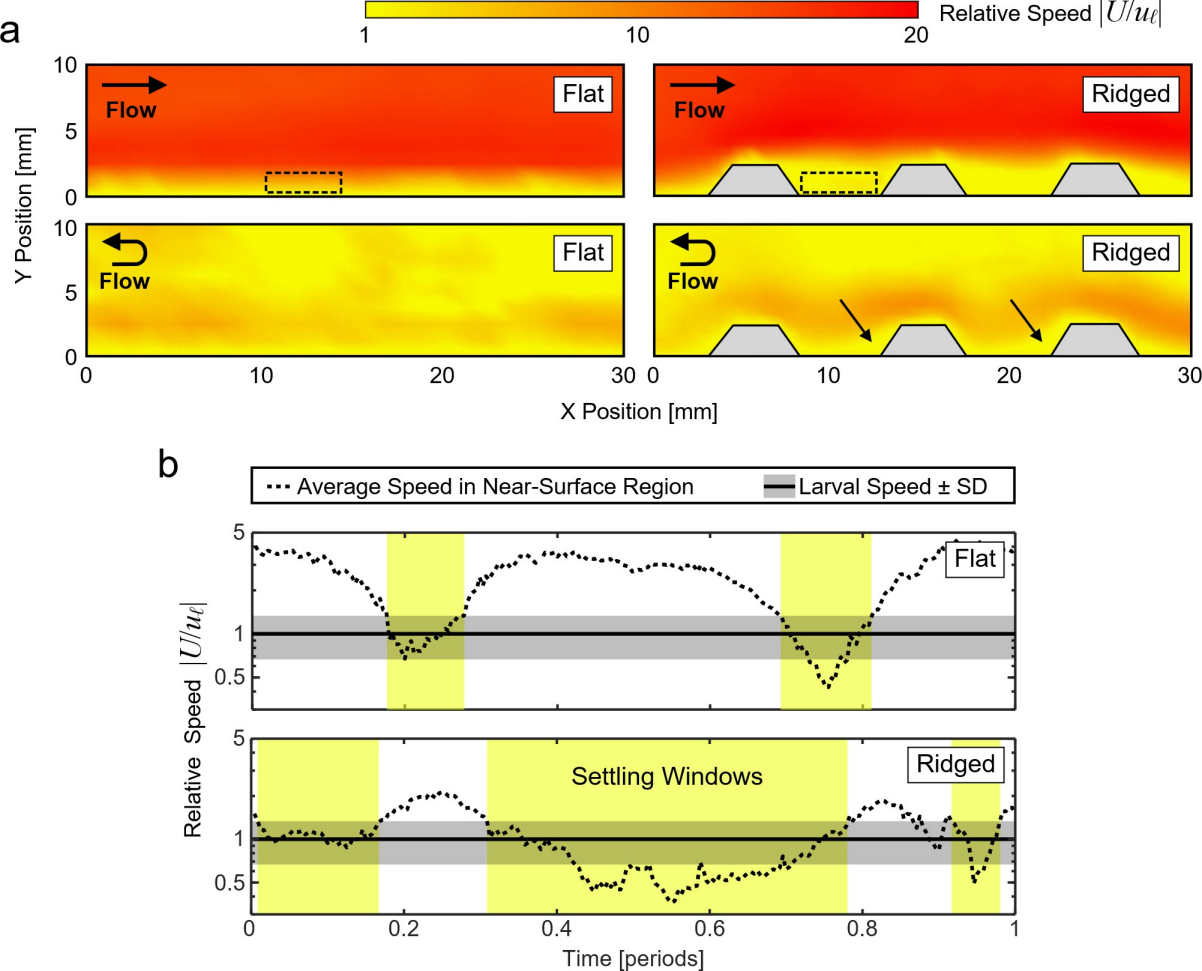
Millimeter-scale ridges increase the duration of larval settling windows. (a) Visualization of the relative fluid speed over flat (*left*) and ridged (*right*) substrates during peak (*top*) and turning point (*bottom*) flow. The fluid velocity (*U*) is normalized by the average larval swimming velocity (*u*_*ℓ*_). Dotted black boxes represent regions ≤1.5 mm above the substrate surface that were used to calculate duration of settlement windows. Black arrows near the bottoms of the ridges indicate regions where the velocity remains low even at the turning points. (b) The average relative flow speed within the dotted black regions plotted over an average period for flat (*top*) and ridged (*bottom*) substrates. The yellow regions highlight the settling windows during which the local flow speed drops below *u*_*ℓ*_ (black line) plus one standard deviation (grey band).

In addition to modifying recirculation in the boundary layer, substrate topography also directly influenced fluid speed near the substrate surfaces (Fig 3A). Fluid speeds were extracted from PTV data and compared to the average swimming speed of coral larvae to reveal regions in the flow where larvae would either be expected to undergo passive transport or would be capable of swimming against the flow. We used a species-averaged larval swimming speed (|*u*_*ℓ*_|) of 3 ± 1 mm s^-1^ (mean ± standard deviation), which was computed using PTV data obtained from swimming *D*. *labyrinthiformis* larvae and previously-reported data on larval swimming speeds (S1 Table in S1 File) [34].

During the peak flow phase, in regions ≥3 mm above both substrate types, the local flow speed (|*U*|) reached up to 60 mm s^-1^, i.e., up to 20 times faster than the average larval swimming speed (|*u*_*ℓ*_|, Fig 3A, *top*). The flow speed generally decreased closer to the substrates, but the thickness of these lower velocity regions varied between substrate types. For ridged substrates, the average local flow speeds in regions up to 3 mm above the substrate were much lower than in the free stream above, ranging from less than 1 to 10 times the larval swimming speed. In contrast, this low velocity region was much thinner over flat substrates, extending only up to 0.75 mm above the substrate.

During the turning point flow phase, regions far above (≥5 mm) the surfaces of both substrate types also had fluid speeds equal to or less than the average larval swimming speed (i.e, |*U*| ≤ |*u*_*ℓ*_|). Closer to the substrate surface (≤1.5 mm), the turning point flow speed was slightly increased over ridged substrates and remained low over flat substrates. However, due to the rapid fluid acceleration before and after the turning point, these regions of low flow speed were transient and short-lived.

Not only was fluid speed near the substrates highly variable during an oscillation period, this variation was strongly influenced by substrate topography (Fig 3B). The fluid speed in the region ≤1.5 mm above the substrate, which for coral larvae is approximately 3 to 5 body lengths, is considered to be crucial for larval settlement [53]. For flat substrates, the flow speed in this region varied by an order of magnitude, ranging from 1.5 to 15 mm s^-1^ during an oscillation period, or 0.5 to 5 times the average larval swimming speed, with the lowest flow speed occurring during the turning point phase. For ridged substrates, the flow speed in this region ranged from 1.2 to 6 mm s^-1^, or 0.4 to 2 times the average larval swimming speed, with the lowest flow speed occurring during the peak flow phase.

To further illustrate the effects of substrate topography on the dynamics of local flow speed, and consequent opportunities for larval settlement, we quantified the duration of the larval settling windows that occurred in the regions ≤1.5 mm above each substrate type (Fig 3B, highlighted regions). Settling windows were defined as time intervals during which |*U*| dropped below |*u*_*ℓ*_| so that larvae could actively swim toward and attach to a substrate [54]. The flat substrates produced only two brief settling windows, each lasting 0.6 s or ~11% of an oscillatory period, at the turning points of the flow (Fig 3B). Ridged substrates, however, produced settling windows that were ~3 times longer (1.86 s each). In fact, in the regions between the substrate ridges, the two settling windows comprised the majority of the flow period (~69% or 3.7 s). In addition to producing larger regions of low flow speed and longer settling windows, the ridged substrates also created small, permanent regions of low velocity at the base of each ridge (Fig 3A, *black arrows*). Even during intervals of high relative flow speed, which did not satisfy the condition of a settling window, the local fluid velocity near the base of the ridges remained equal to or less than the average larval swimming speed. Notably, these regions corresponded to the locations where larval settlement was the highest in the oscillatory flow experiments (Fig 1E).

### Agent-based simulations of larval transport and settlement further demonstrate the effects of substrate topography

**Fig 4:**
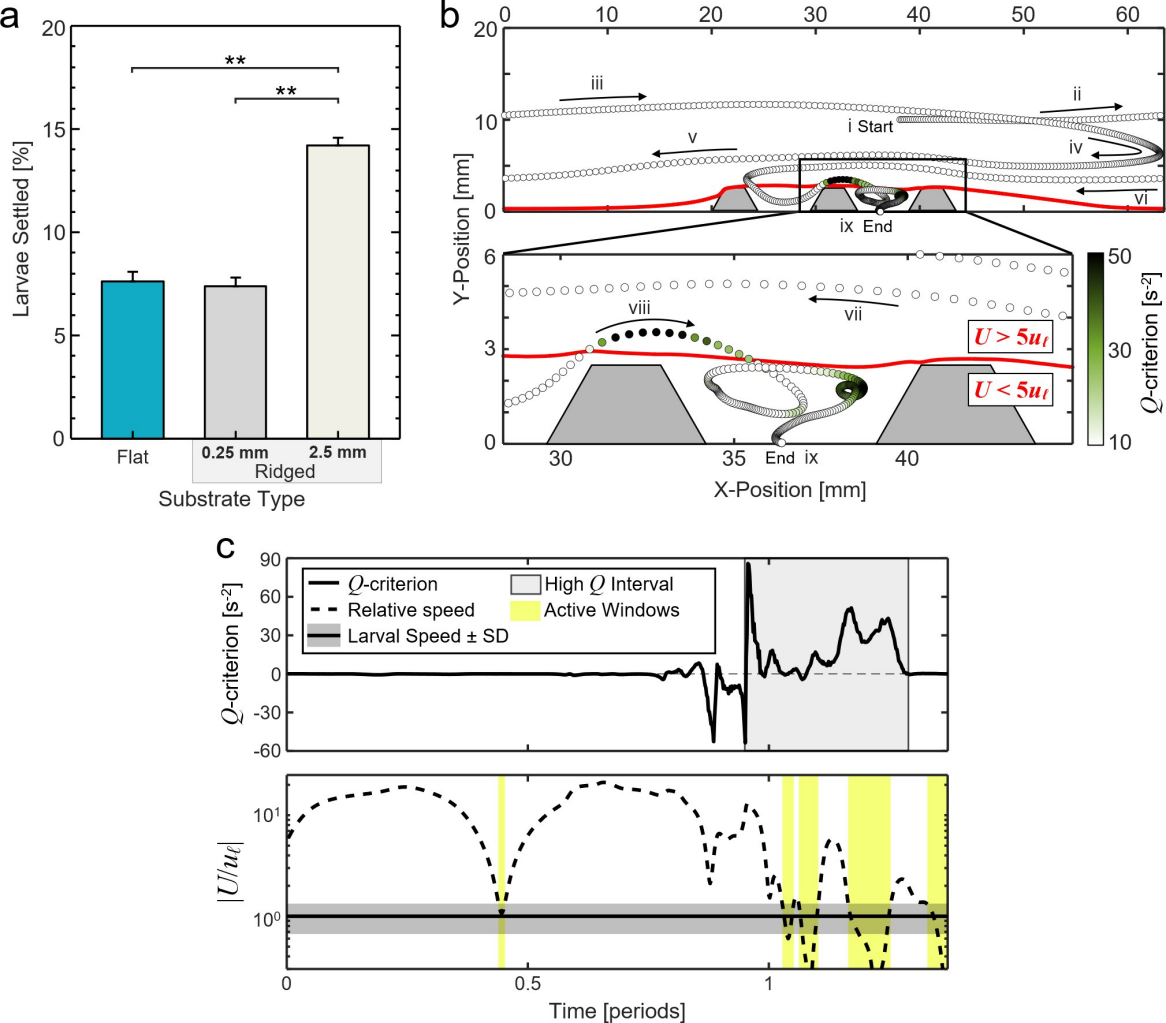
Millimeter-scale ridges modify the boundary layer flow to facilitate larval transport and settlement. (a) Results of larval settlement simulations on flat substrates and ridged substrates with millimeter-scale (2.5 mm) and sub-millimeter-scale (0.25 mm) ridges in oscillatory flow. There were significant differences in settlement between substrates with millimeter-scale ridges and flat and sub-millimeter-scale ridged substrates (**: *p* < 0.001; post-hoc Tukey HSD). (b) Simulation of a larva trajectory (colored dots) over a surface with millimeter-scale ridges. The color of the dot indicates the instantaneous *Q*-criterion experienced by the larva at each location. The spacing between dots increases with larval speed. The red line shows the boundary between regions of *U* > 5*u*_*ℓ*_ and *U* < 5*u*_*ℓ*_ during the peak rightward flow phase. (c) The instantaneous *Q*-criterion (*top*) and relative flow speed (*bottom*) experienced by the simulated larva in (b). After encountering multiple regions with high-*Q* values (grey band), the larva experiences several intervals of low relative fluid velocity, or active windows (yellow bands), before settling.

In agreement with flume settlement experiments, agent-based larval simulations showed approximately double the rate of settlement onto ridged substrates than onto flat substrates (Fig 4A). Average settlement onto ridged substrates was 14.2% compared to 7.6% onto flat substrates. To probe the sensitivity of this phenomenon, an additional simulation was run using substrates with ridges that were 0.25 mm tall and spaced 0.75 mm apart, length scales similar to the larval length scale that would be expected to increase settlement according to Attachment Point Theory. Surprisingly, larval settlement in these simulations was nearly identical to in simulations of flat substrates, with the 0.25 mm ridges receiving an average of 7.4% settlers (Fig 4A). These results prompted us to further investigate the specific trajectories of simulated larvae and the local hydrodynamic environments that produced double the average rate of settlement onto the 2.5 mm ridged substrates compared to the other substrate types.

Larval transport and settlement over 2.5 mm ridged substrates is illustrated by the representative trajectory of a single simulated larva shown in Fig 4B. Initially, this simulated larva was seeded in the flow 1 cm above the substrate (Fig 4B(i)). The larva was then transported by the oscillatory flow, traveling large distances horizontally while slowly approaching the substrate surface (Fig 4B(ii—vi)). As the larva neared the substrate, it was directed toward the surface over a ridge (Fig 4B(viii)). Finally, the larva contacted the substrate surface with a net speed that was lower than the larval swimming speed plus one standard deviation, and the larvae was therefore considered to be settled (Fig 4B(ix)). Upon closer inspection of the trajectory before settlement, the larva traversed through a region of high rotation in which the *Q*-criterion was above the threshold value, *Q*_*thresh*_ (Fig 4B, lower, magnified panel). Immediately following, the larva was transported from a high-velocity flow region of *U > 5u*_*ℓ*_ to a low-velocity flow region of *U < 5u*_*ℓ*_ between the ridges.

The hydrodynamic mechanisms that allowed larval settlement were further elucidated by computing the instantaneous *Q*-criterion and relative swimming speed experienced by the simulated larva along its trajectory. The instantaneous *Q*-criterion fluctuated rapidly and depended on the location of the larva in the flow with respect to the substrate surface (Fig 4C, *top*). In the bulk flow region, velocity gradients were minimal and therefore the larva experienced *Q*-criterion values close to zero. Near the surface or a ridge, the larva experienced large negative (strain) or positive (rotation) *Q*-criterion values, respectively. Finally, very close to the substrate surface, the larva experienced a low *Q*-criterion value, which facilitated settlement.

The instantaneous relative flow speed experienced by the larva fluctuated slowly at first due to the length of the oscillatory flow period, and more rapidly as the larva approached the surface (Fig 4C, *bottom*). Tracking of the flow speed enabled the identification of “larval active windows,” defined as intervals during which the larval swimming speed was greater than the local instantaneous fluid speed, i.e., when *U/u*_*ℓ*_ < 1. In the bulk flow region, the relative flow speed was high (*U/u*_*ℓ*_ ≫ 1) except for short intervals during the flow turning points. Closer to the substrate, the relative flow speed fluctuated rapidly between high and low values, and the larva experienced multiple consecutive active windows upon arriving between the ridges (Fig 4C, *bottom*, S4 Video). After experiencing a final active window close to the substrate surface, the larva was able to settle; this occurred when a larval active window coincided with a settling window ≤1.5 mm from the substrate. Conversely, for simulated trajectories over flat substrates and substrates with 0.25 mm ridges, larvae experienced high shear and strain (reorientation) and fewer larval active windows, therefore preventing high levels of settlement (S3-S5 Figs in S1 File).

## Discussion

The importance of hydrodynamics in larval navigation and settlement has been recognized for some time. Studies have shown that changes in wave intensity can induce larvae to swim upward or downward [55] and high shear forces can trigger settlement competency [56, 57]. As well, specific flow regimes can inhibit recruitment because most larvae have a limited ability to swim against high velocity fields [34, 58] or to settle in the presence of strong oscillatory flows [33, 43], which are often found in the benthic boundary layer (BBL) over reef canopies [36, 59]. Despite the known effects of substrate topography on BBL flow, studies of coral larval settlement are still often conducted in static conditions, and many of the flow-based studies that do exist utilize complex or poorly controlled topographies, which complicates the interpretation of results. Furthermore, in part due to their rarity and practical difficulties in their use, live motile larvae are not utilized in many studies of settlement hydrodynamics [33, 59, 60]. Thus, the direct link between substrate surface topography, BBL flow, and coral larval settlement remains elusive, yet it is paramount to improve the effectiveness of reef restoration and recovery efforts.

We addressed this knowledge gap by investigating coral larval settlement on engineered restoration substrates under controlled oscillatory flow. Experiments with *D*. *labyrinthiformis* and *C*. *natans* larvae revealed two ways in which BBL flows over millimeter-scale ridges enhanced settlement compared to flat surfaces. First, the interaction of the flow and the substrate ridges produced recirculation zones that directed larvae from regions of high velocity–in which they are effectively passive particles–to regions of low velocity where they could actively move in relation to the surrounding fluid. Here, the utilization of particle tracking methods and the *Q*-criterion metric of vortex identification allowed us to locate and quantify transient regions of recirculation that formed over larval settlement locations (Fig 2). Second, millimeter-scale substrate ridges extended the settling window durations in the near-surface flows by keeping velocities equal to or less than the average coral larval swimming speed plus one standard deviation. The duration of these settling windows for ridged substrates was extended by at least 300% compared to flat substrates (Fig 3). These two experimental observations were reinforced by an agent-based simulation, which demonstrated that the combination of fluid recirculation and extended settling windows over ridged substrates created more opportunities for coral larvae to actively navigate toward and settle onto substrate surfaces (Fig 4). Additionally, the generality of these simulation results was ensured by using a species-averaged larval swimming speed.

Our combined experimental and modeling study illustrates the importance of often-overlooked, millimeter-length substrate features in marine larval settlement. Most reef-scale bathymetric mapping does not quantify topography below a resolution of ~10 cm [61–63], while most topography-based settlement studies have focused on the effect of micro-scale features of <1 mm [24, 64]. According to Attachment Point Theory, larvae generally settle within topographical features close to their size (~0.1–1 mm) [25]. However, because most controlled larval settlement studies have been performed in static or low-flow conditions [65], they do not fully mimic the complex physical environment in which coral larvae must settle in nature. In our simulations, larval settlement onto surfaces with 0.25 mm tall ridges was not statistically different than settlement onto flat surfaces. We did not detect sustained periods comprising any of the key hydrodynamic characteristics associated with increased settlement (e.g., vorticity, rotation) over either of these substrate types in our flume experiments. Therefore, while sub-millimeter-scale topography helps promote larval settlement in low-flow and static conditions, it is unlikely to do so in most wave-dominated, natural reef environments. In the future, these settlement trends should also be investigated for a range of flow conditions and for topographical features of varying spacing to height ratios, which have been shown to have an effect on BBL flow and heat and mass transfer between the substrate and water column [66].

To date, coral reef restoration has not been heavily focused on promoting natural coral settlement–particularly in strongly wave-dominated conditions–but this may be possible in the future, especially by building upon and leveraging the increasing diversity of established methods for fostering larval settlement [67]. Traditionally, most laboratory-reared coral juveniles were pre-settled onto substrates under low-flow or static conditions. More recently, restoration approaches have expanded to include direct larval seeding [68] and *in situ* settlement pools [9, 69], which both achieve larval settlement under natural flow regimes that have been dampened somewhat by the seeding/settlement structures themselves. These approaches have revealed essential details about the materials, substrate communities, and benthic communities that allow settlement and survival. Now, insights and innovations gained from these approaches can be combined with hydrodynamic modeling to engineer high-performance substrates designed to attract and entrain larvae, foster settlement, shelter early post-settlement juveniles, promote calcification, and support the dominance of corals relative to other benthic competitors, even in wave-dominated, nearshore environments. Some progress has already been made in this area, e.g., the installation of topographically complex concretes to promote recruitment to seawalls and breakwaters [70]. Nevertheless, facilitating the robust settlement of natural populations of coral larvae remains a growth opportunity for materials engineering and fluid physics.

Here, we focused on engineered substrates and their potential applications in coral reef restoration, but our results also have relevance to the failure of natural coral recruitment that has been observed worldwide [1–3]. It is widely accepted that coral reefs have undergone a topographic flattening at the centimeter to meter scale [71–73]. Similar, but less appreciated, is the flattening of coral reefs that has occurred at the millimeter to centimeter scale as a consequence of coral loss and algal overgrowth. The majority of now-dominant benthic groups (e.g., turf algae, macroalgae, sponges, and cyanobacteria) form little to no rigid structure at these scales. Yet, these are precisely the type of topographic structures that facilitate coral settlement, millimeter-scale structures that were once formed by bare coral skeletons, CCAs, bivalves, and intense parrotfish and urchin grazing on hard substrates. Here, we have shown that the loss of such topography has a profound effect on the hydrodynamics of larvae settlement, and hence, the restoration of such topography may help to reverse declining recruitment rates in conjunction with efforts to ensure larval supply and protect recruits and juveniles post-settlement, e.g., by combatting rising sea temperature-induced bleaching events [74–76].

## Supporting information

S1 File Supplementary material to the manuscript that contains 9 figures and 1 table. (DOCX)

S1 Video Tracer particles in flow over a flat CaCO_3_-based settlement substrate. Recorded at 90 fps and played in real time.
(MP4)

S2 Video Tracer particles in flow over a ridged CaCO_3_-based settlement substrate. Recorded at 90 fps and played in real time.
(MP4)

S3 Video Recirculation over a 3D printed ridged substrate identified by computing the *Q*-criterion from particle tracking velocimetry (PTV) data. *Q*-criterion values are averaged over 0.22s intervals.
(MP4)

S4 Video Active windows a simulated larva experiences before settling. The larva turns yellow when experiencing an active window. Created at 100 fps and played at 1/5 speed.
(MP4)

S5 Video Tracer particles in flow over a 3D printed ridged substrate. Recorded at 90 fps and played in real time.
(MP4)
