# Supplementary material for: Millimeter-scale topography facilitates coral larval settlement in wave-driven oscillatory flow
Source: PLoS One. 2022 Sep 12;17(9):e0274088. doi: 10.1371/journal.pone.0274088 (PMC9467362; doi:10.1371/journal.pone.0274088)
Supplement: S1 File — (DOCX) [file pone.0274088.s001.docx]

**Supplementary Information:**

Millimeter-scale topography facilitates coral larval settlement in wave-driven oscillatory flow

Mark A. Levenstein,^1,2†^ Daniel J. Gysbers,^3^ Kristen L. Marhaver,^4*^ Sameh Kattom,^1^

Lucas Tichy,^4,5^ Zachary Quinlan,^6^ Haley M. Tholen,^1^ Linda Wegley Kelly,^6^ Mark J. A. Vermeij,^4,7^ Amy J. Wagoner Johnson^1,2,8*^ and Gabriel Juarez^1*^

**Additional Experimental Methods**

***Larval Rearing***

Larvae were reared following previously-published methods [1]–[4]. Briefly, gamete bundles were mixed in 1 L polycarbonate fat separators within 1 hour of collection to allow fertilization. After 60 minutes, fertilized embryos were rinsed 6 times with 0.5 µm filtered sea water to remove excess sperm (FSW; 25.4 cm × 6.35 cm stacked sediment filters, spun polypropylene, pore sizes 50 µm, 20 µm, 5 µm, and 0.5 µm; H_2_O Distributors, Marietta, GA). Developing embryos were transferred to 1 L clear polystyrene clamshell food containers containing approximately 800 mL FSW and left overnight to develop into larvae. Swimming larvae were separated from unfertilized eggs and lipid detritus by siphoning or by rinsing larvae over a 150 µm nylon mesh filter. Water changes and container changes were performed approximately every 2 days to maintain larval health and motility prior to use in experiments.

***Flume Tank Design***

The flume had a central, 36 cm long transparent section made from acrylic with a square 10 × 10 cm^2^ cross-sectional area, allowing visualization of the flows within using a camera and laser sheet. This acrylic section was connected on one side to a vertical 25.4 cm long 10 cm diameter polyvinyl chloride (PVC) arm using a 3D-printed square-to-circular pipe connector and a PVC elbow. The arm was topped with a 3D-printed cap that served as a mount for the driving motor. The other side of the central acrylic section was connected to another vertical PVC arm using a 3D-printed connector and a PVC T-socket. The PVC socket was sealed on the unconnected side with an expansion plug that was removed in order to drain the flume. Settlement substrates were introduced into the flume by disconnecting the PVC arms from the 3D-printed connectors on both sides of the acrylic section. These connections were reversibly sealed by nitrile O-rings. A DC gearmotor (McMaster-Carr) was mounted to the flume cap to drive the oscillatory flow. The motor rotated a drive wheel that was attached to the piston shaft with a 3D-printed, custom scotch yoke mechanism fitted through a slot in the cap. The period of oscillation was adjusted by changing the voltage supplied to the gearmotor, and therefore the rotation speed of the drive wheel, using a micro-controller. All the 3D-printed parts were made of polylactic acid (PLA), and the inner surfaces were coated in clear-cast epoxy (Alumilite) to prevent water from permeating the parts. A wood frame was constructed around the flume for increased stability and rigidity during operation and transport.

***Settlement Substrate Fabrication***

Settlement substrates were prepared using an un-aged lime mortar made from a 1:9:10 mixture (by mass) of glass fibers (1/32'' milled, Fibre Glast), kalkwasser powder (ESV Aquarium Products), and fresh water, respectively. For ease of handling and compatibility with other studies, the substrates were molded into small disks resembling the rounded ceramic tiles used in many coral studies and propagation activities (i.e., “frag plugs”). The mortar was cast in silicone rubber molds to produce disks with either a flat top surface or bearing a regular array of ridges 2.5 mm in height. After overnight casting, substrates were removed from the silicone molds and carbonated in a high CO_2_ environment for 1 – 7 days. During this time, the carbonation process converted the mortar into CaCO_3_ to harden the substrates [5]. Substrates were then conditioned in a flow-through aquarium system with raw seawater for 4 days to de-gas pores and remove unreacted lime (Ca(OH)_2_) immediately prior to settlement experiments.

***Larval Settlement Experiments***

Larval settlement experiments began two days after spawning, and larvae were used in the flume up to 1 week after they first displayed motility. The experimental procedure was as follows. First, the flume was partially filled with filtered seawater (FSW). Subsequently, an initial stock of 750 – 1000 larvae was introduced into the flume using a funnel and hose inserted through the uncapped PVC arm. Additional FSW was added to ensure proper fill height and to prevent the formation of an air gap between the piston head and the water. This resulted in a final larval density between 50 – 70 larvae per liter of FSW in the flume. Experiments were conducted in a room where the temperature was maintained at 27.5 ± 1.0 °C to match the temperature of the reef where gametes were collected. The motor was then connected to the micro-controller, and the piston speed was set to produce the desired flow period. After each experimental run, larval settlement positions were recorded, the water was drained, and unattached larvae were released into the sea. Between each individual run, the flume was cleaned with a jet of fresh water, rinsed with FSW, and drained. Between each round of experiments with a particular species, the flume was filled with fresh water and left to soak in the sun for 2 – 3 days.

**Supplementary Video Descriptions**

***Video S1*** – Tracer particles in flow over a flat CaCO_3_-based settlement substrate. Recorded at 90 fps and played in real time.

***Video S2*** – Tracer particles in flow over a ridged CaCO_3_-based settlement substrate. Recorded at 90 fps and played in real time.

***Video S3*** – Recirculation over a 3D printed ridged substrate identified by computing the *Q*-criterion from particle tracking velocimetry (PTV) data. *Q*-criterion values are averaged over 0.22s intervals.

***Video S4*** – Active windows a simulated larva experiences before settling. The larva turns yellow when experiencing an active window. Created at 100 fps and played at 1/5 speed.

***Video S5*** – Tracer particles in flow over a 3D printed ridged substrate. Recorded at 90 fps and played in real time.

**Supplementary Figures**


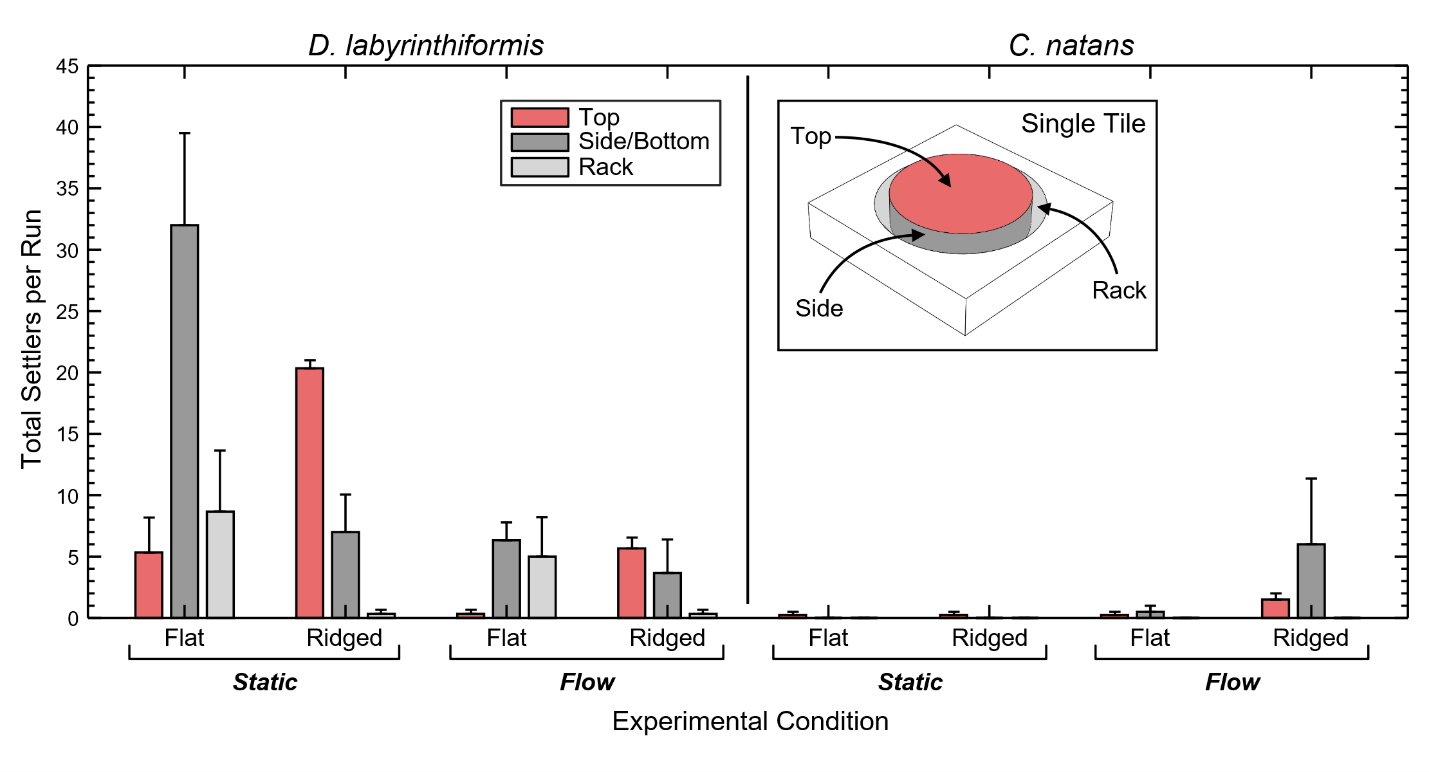


**Figure S1:** Raw data from *D. labyrinthiformis* and *C. natans* larval settlement experiments on flat and ridged substrates under both oscillatory flow and static conditions. Each bar extends to the mean number of settlers and the error bars denote the standard error of the mean (*n* = 3 replicate runs). The inset illustrates how settlers were scored based on their settlement location: top, side (and bottom), and rack.


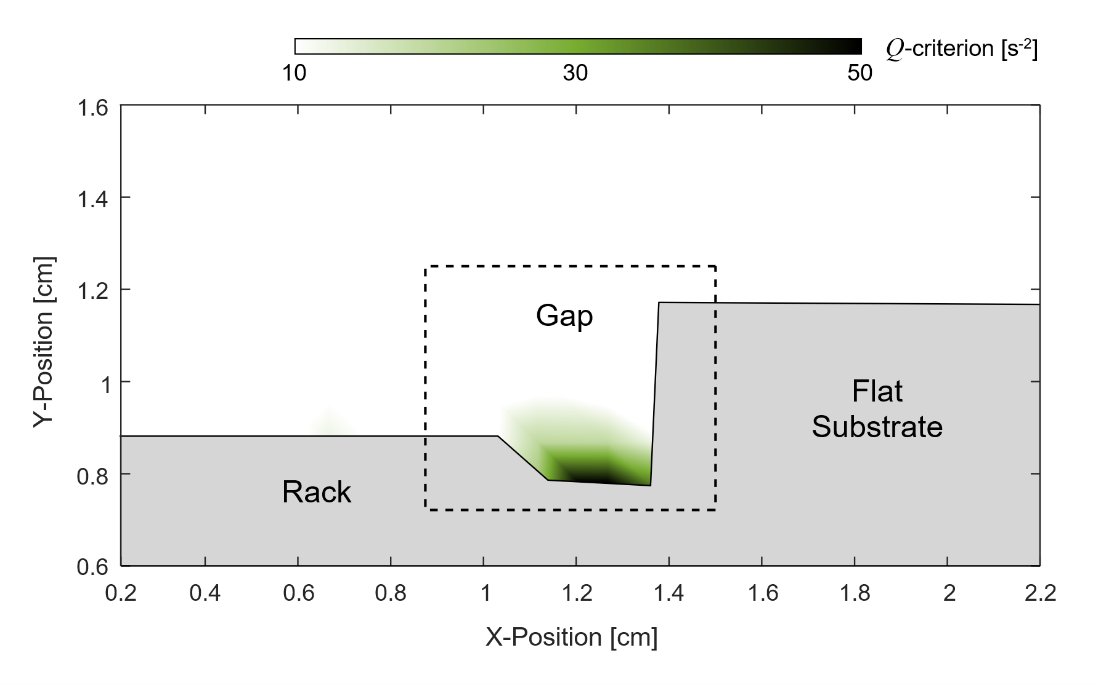


**Figure S2:** Regions of flow recirculation identified over the gaps next to flat substrates during the turning point flow phase. Recirculation was identified using the *Q*-criterion metric.


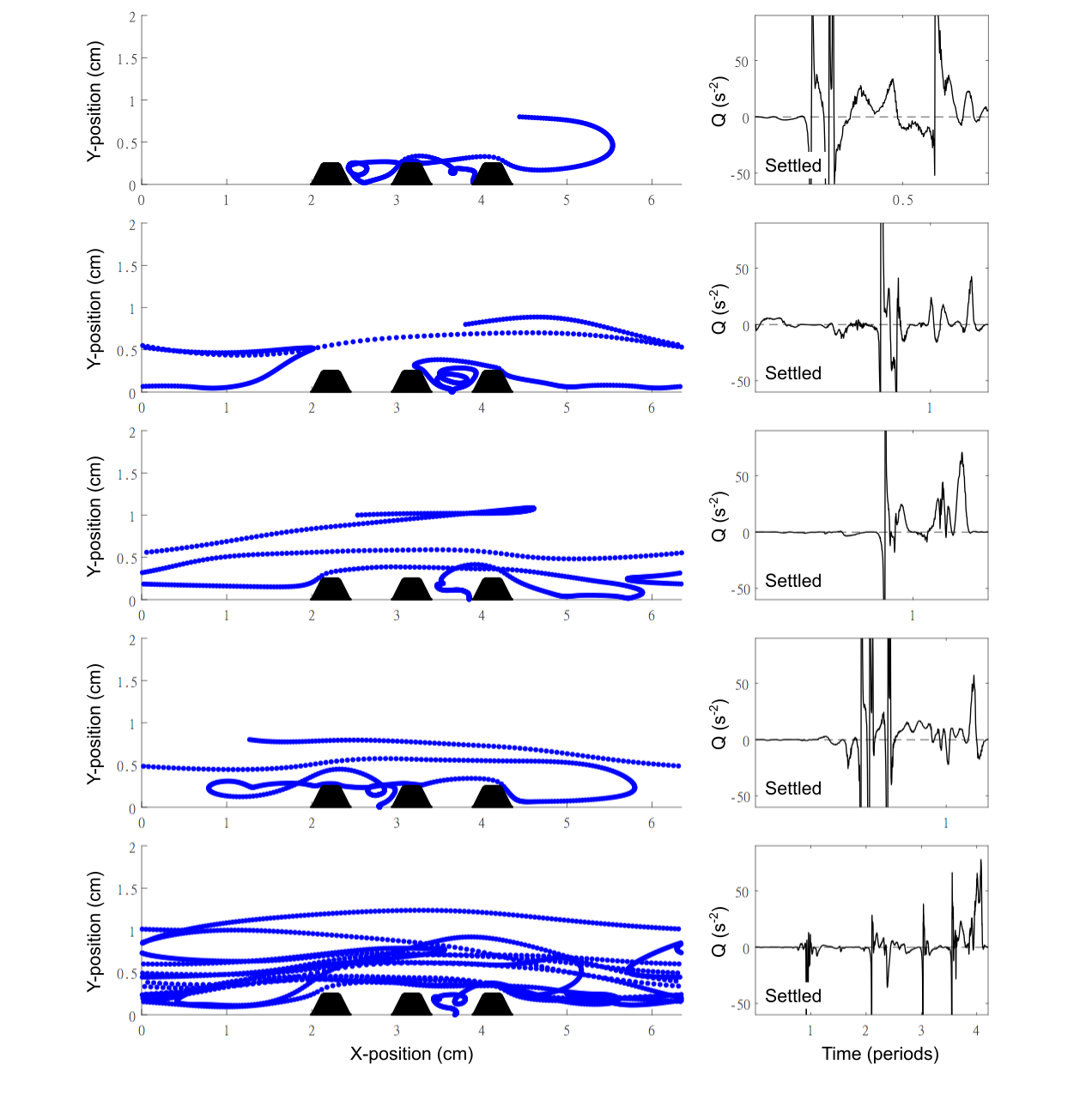


**Figure S3:** Example simulated larval paths and *Q*-criterion values over the 2.5 mm ridged substrate topography.


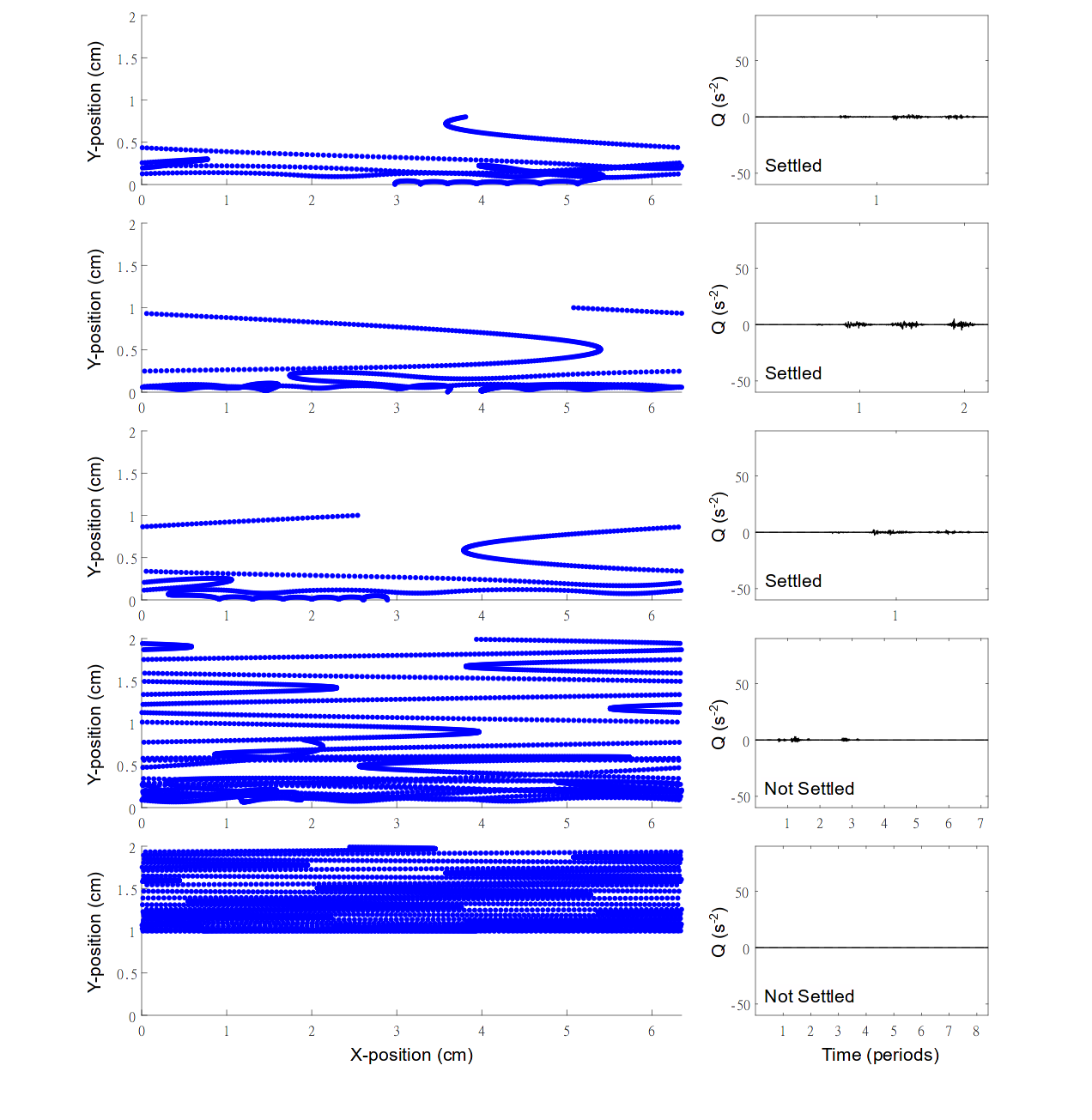


**Figure S4:** Example simulated larval paths and *Q*-criterion values over the flat substrate topography.


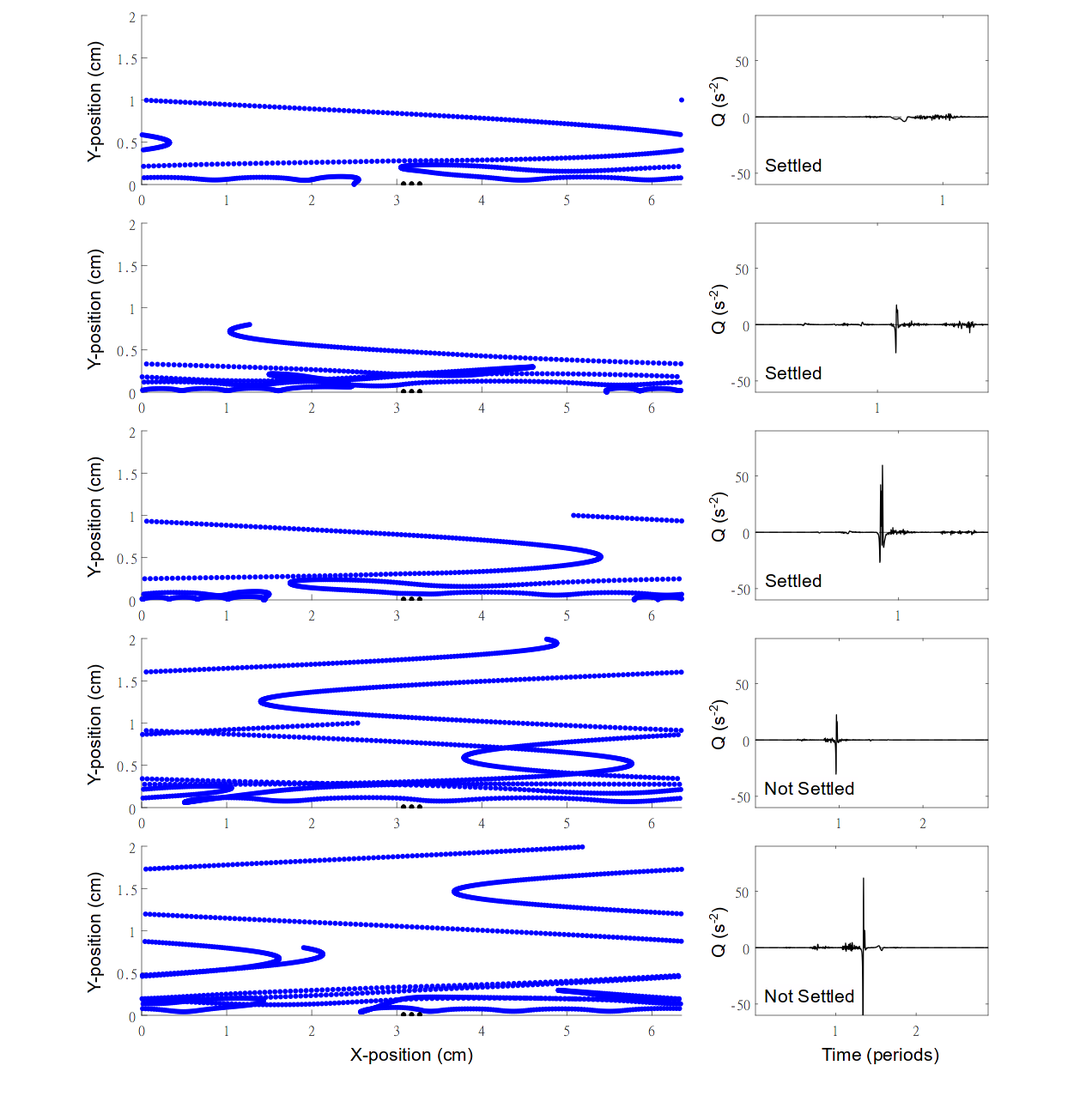


**Figure S5:** Example simulated larval paths and *Q*-criterion values over the 0.25 mm ridged substrate topography.


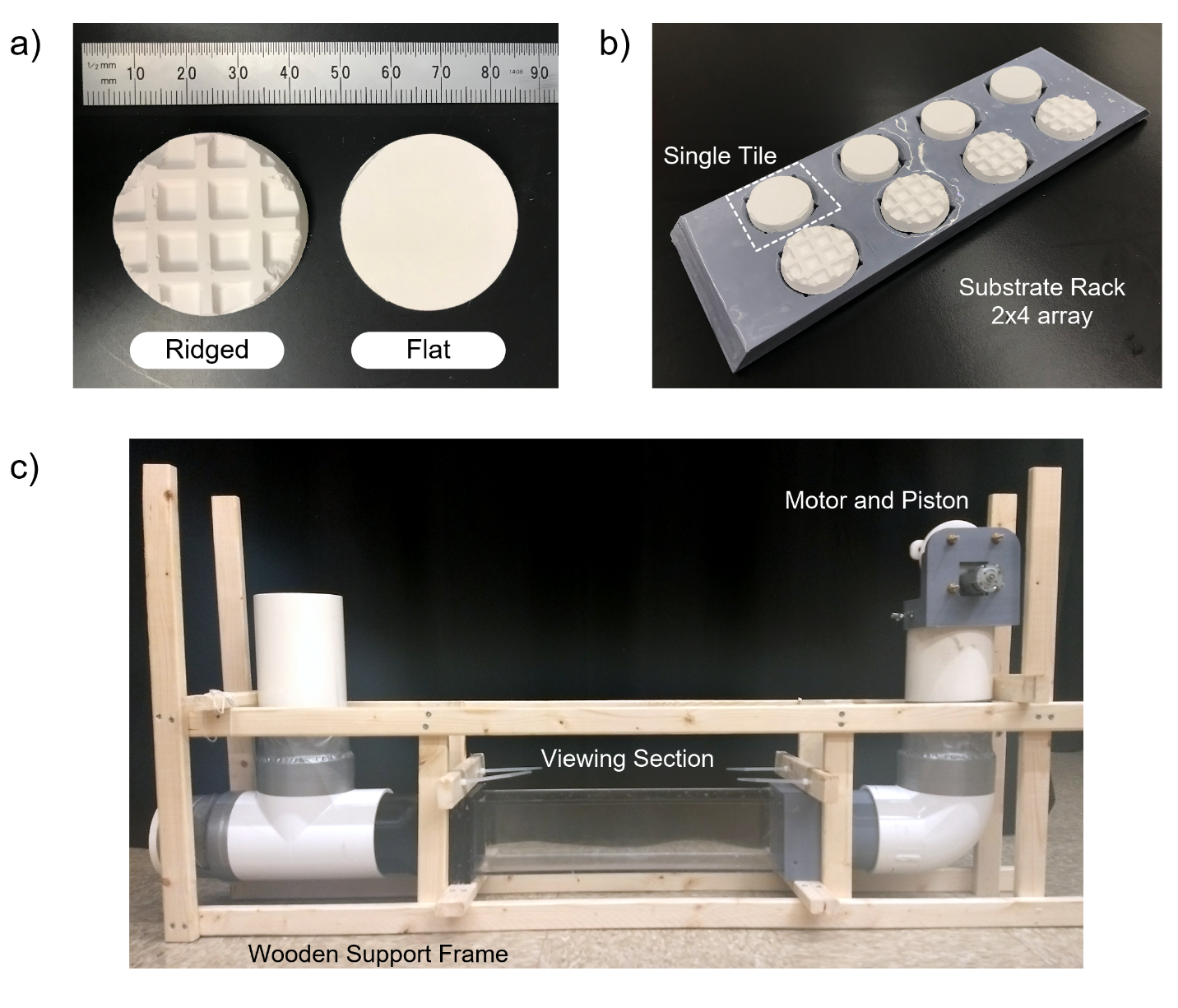


**Figure S6:** (a) Photograph of flat and ridged CaCO_3_ settlement substrates. (b) Photograph of substrates loaded into the 3D-printed rack that was used to hold substrates in the flume tank during settlement experiments. (c) Photograph of the flume tank with the wooden support frame.


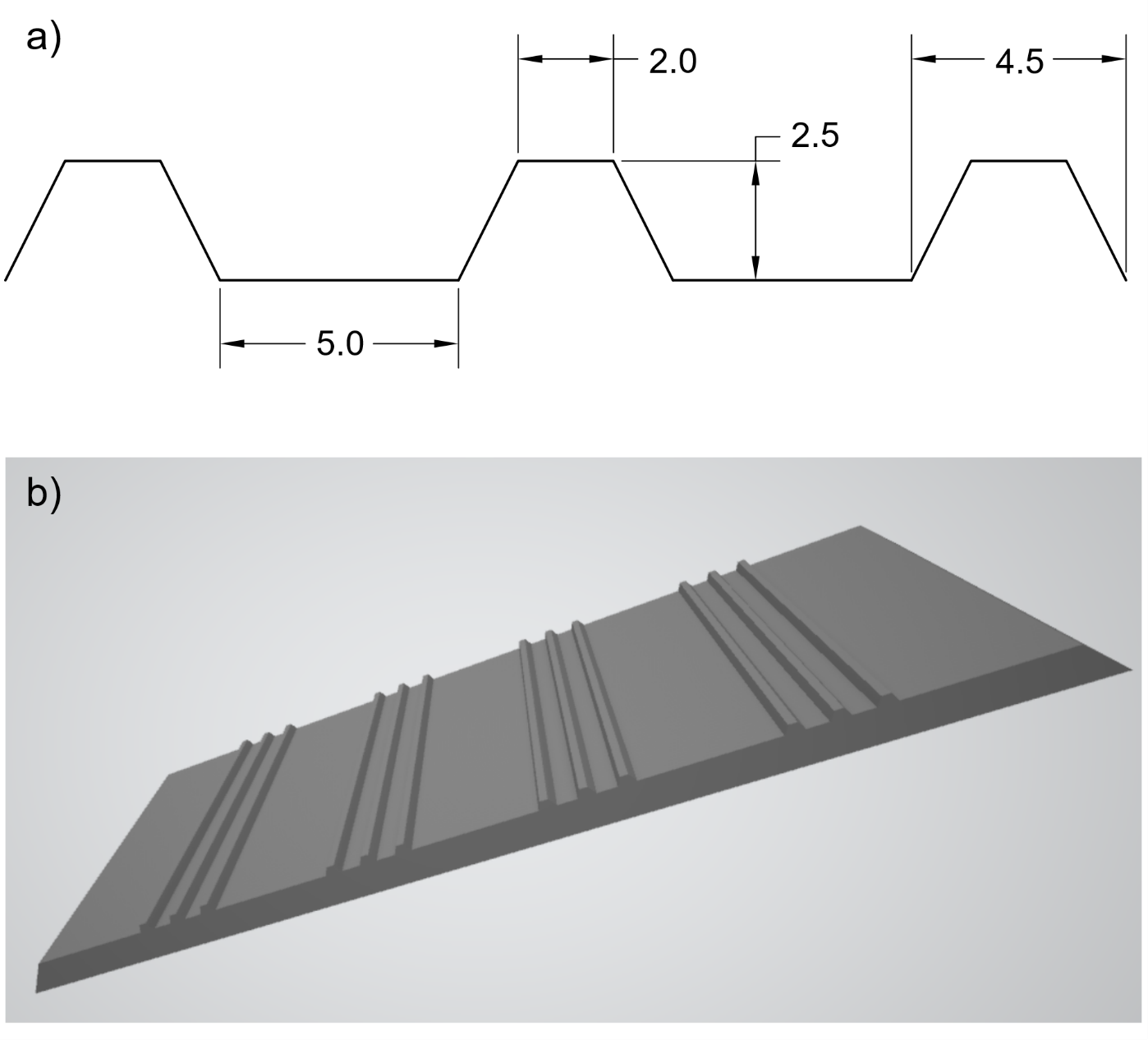


**Figure S7:** (a) 2D profile of the ridged CaCO_3_ substrates used to make the ridged model for flow simulations and the (b) 3D-printed ridged substrate for flow visualization studies. All dimensions are given in millimeters.


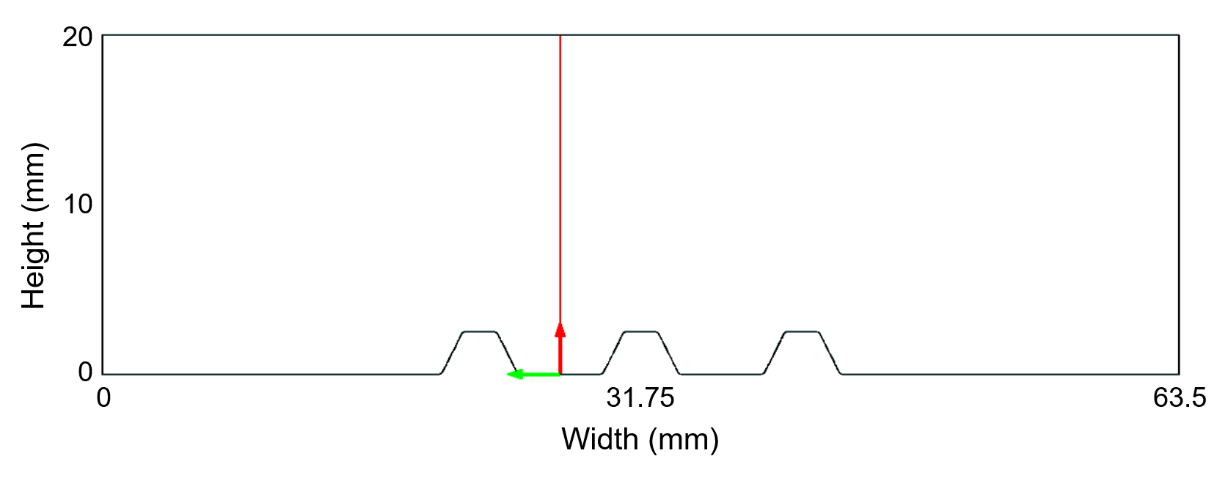


**Figure S8:** Ridged substrate model used for COMSOL simulations of oscillatory flow.


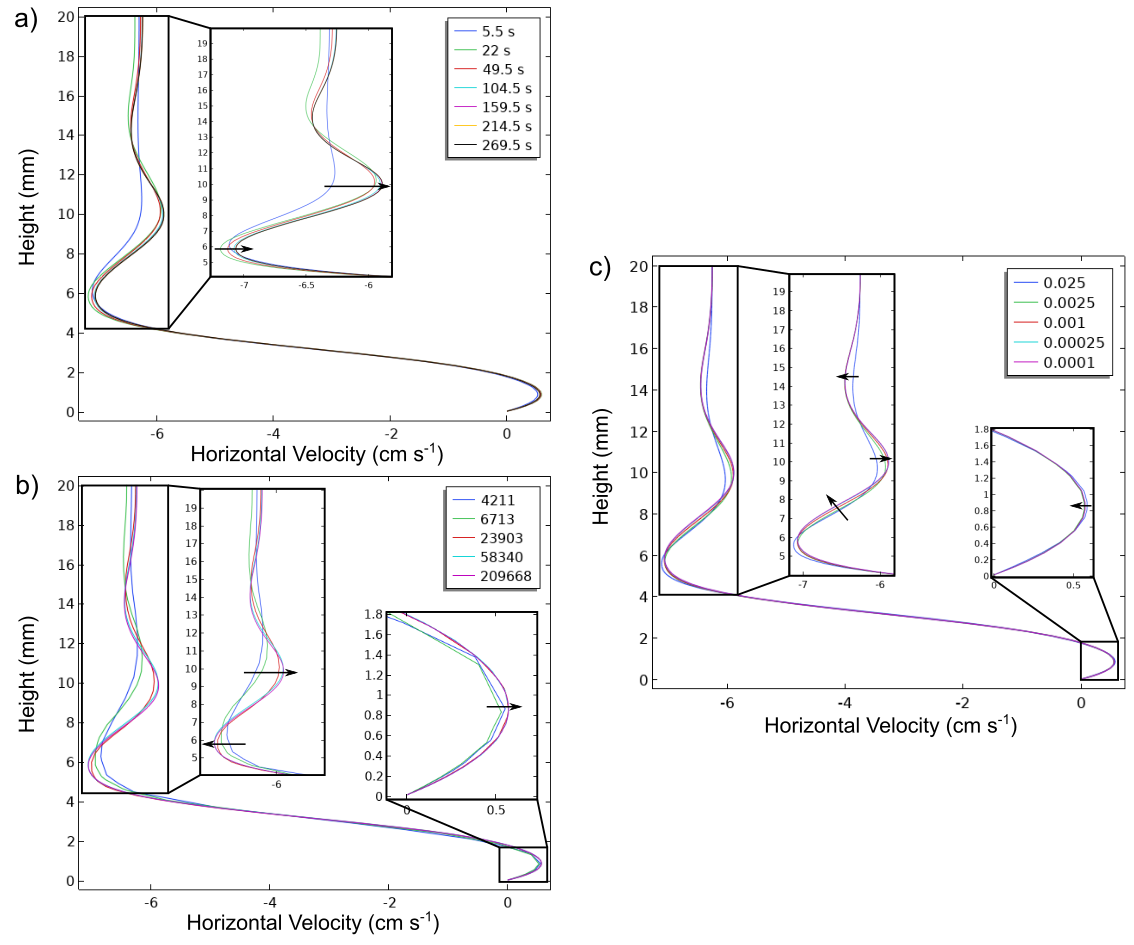


**Figure S9:** Validation of the COMSOL model and simulation showing (a) the achievement of periodicity, (b) the convergence of the mesh, and (c) the convergence of relative tolerance. These parameters were evaluated using the velocity profile between to ridges (red line, Figure S8) during peak flow to the left (at the start of the 5.5 s oscillatory period). The velocity profile began to reach periodicity during the 30^th^ period (159.5 s), and we utilized the flow field simulated after 50 periods (269.5 s) for agent-based larval modeling. The velocity profiles converged with a mesh containing 58340 elements, and this mesh was utilized for all subsequent simulations. The adaptive solver in COMSOL modifies the time step to minimize the error according to a relative tolerance. The profiles converged at a relative tolerance of 0.00025, which was used for all simulations. The black arrows illustrate the convergence of the velocity curves toward the true velocity profile.

**Supplementary Table**

**Table S1:** Larval swimming data used in calculating the average larval swimming speed, |*u_ℓ_*|

| **Species** | **Direction** | **Mean Speed (mm/s)** | **Reference** |
| --- | --- | --- | --- |
| *Heliofungia actiniformis* | Horizontal | 1.57 | [6] (internal ref. 46) |
|  | Up | 1.66 |  |
|  | Down | 2.76 |  |
| *Pocillopora damicornis* | Horizontal | 1.90 | [6] (internal refs. 34 and 47) |
|  | Up | 2.79 | [6] (internal ref. 51) |
|  | Down | 4.79 |  |
| *Coelastrea aspera* | Horizontal | 2.73 | [6] (internal ref. 48) |
| *Agaricia tenuifolia* | Up | 2.10 | [6] (internal ref. 49) |
|  | Down | 3.60 |  |
| *Galaxea horrescens* | Up | 2.41 | [6] (internal ref. 50) |
|  | Down | 3.86 |  |
| *Porites astreoides* | Up | 2.8 | [6] (internal ref. 49) |
|  | Down | 4.3 |  |
| *Isopora bruggemanni* | Up | 2.86 | [6] (internal ref. 52) |
|  | Down | 3.55 |  |
| *Seriatopora hystrix* | Up | 3.33 | [6] (internal ref. 53) |
|  | Down | 4.44 |  |
| *Diploria labyrinthiformis* | Horizontal | 2.10 | This study |
| **Average (\|*u_ℓ_*\|)** | – | 2.98 | – |
| **Standard Deviation** | – | 0.96 | – |

**References**

[1] M. Vermeij, N. Fogarty, and M. Miller, “Pelagic conditions affect larval behavior, survival, and settlement patterns in the Caribbean coral Montastraea faveolata,” *Mar. Ecol. Prog. Ser.*, vol. 310, no. 2, pp. 119–128, Apr. 2006.

[2] K. L. Marhaver, M. J. A. Vermeij, F. Rohwer, and S. A. Sandin, “Janzen-Connell effects in a broadcast-spawning Caribbean coral: Distance-dependent survival of larvae and settlers,” *Ecology*, vol. 94, no. 1, pp. 146–160, 2013.

[3] K. L. Marhaver, M. J. A. Vermeij, and M. M. Medina, “Reproductive natural history and successful juvenile propagation of the threatened Caribbean pillar coral Dendrogyra cylindrus.,” *BMC Ecol.*, vol. 15, no. 1, p. 9, 2015.

[4] V. F. Chamberland, S. Snowden, K. L. Marhaver, D. Petersen, and M. J. A. Vermeij, “The reproductive biology and early life ecology of a common Caribbean brain coral, Diploria labyrinthiformis (Scleractinia: Faviinae),” *Coral Reefs*, vol. 36, no. 1, pp. 83–94, Mar. 2017.

[5] M. A. Levenstein *et al.*, “Engineered Substrates Reveal Species-Specific Inorganic Cues for Coral Larval Settlement,” *ChemRxiv*, pp. 1–25, 2021.

[6] T. Hata *et al.*, “Coral larvae are poor swimmers and require fine-scale reef structure to settle,” *Sci. Rep.*, vol. 7, p. 2249, 2017.
